# A Monovalent Mt10-CVB3 Vaccine Prevents CVB4-Accelerated Type 1 Diabetes in NOD Mice

**DOI:** 10.3390/vaccines11010076

**Published:** 2022-12-29

**Authors:** Mahima T. Rasquinha, Ninaad Lasrado, Meghna Sur, Kiruthiga Mone, Haowen Qiu, Jean-Jack Riethoven, Raymond A. Sobel, Jay Reddy

**Affiliations:** 1 School of Veterinary Medicine and Biomedical Sciences, University of Nebraska-Lincoln, Lincoln, NE 68583, USA; 2 Center for Virology and Vaccine Research, Beth Israel Deaconess Medical Center, Harvard Medical School, Boston, MA 02115, USA; 3 Center for Biotechnology, University of Nebraska-Lincoln, Lincoln, NE 68588, USA; 4 Department of Pathology, Stanford University, Stanford, CA 94305, USA

**Keywords:** vaccine, Coxsackievirus B3, Coxsackievirus B4, insulitis, type 1 diabetes, cross-protection

## Abstract

Enteroviruses, which include Coxsackieviruses, are a common cause of virus infections in humans, and multiple serotypes of the group B Coxsackievirus (CVB) can induce similar diseases. No vaccines are currently available to prevent CVB infections because developing serotype-specific vaccines is not practical. Thus, developing a vaccine that induces protective immune responses for multiple serotypes is desired. In that direction, we created a live-attenuated CVB3 vaccine virus, designated mutant (Mt)10, that offers protection against myocarditis and pancreatitis induced by CVB3 and CVB4 in disease-susceptible A/J mice. Here, we report that the Mt10 vaccine protected against CVB4-triggered type 1 diabetes (T1D) in non-obese diabetic (NOD) mice but the expected subsequent development of spontaneous T1D in these genetically predisposed NOD mice was not altered. We noted that Mt10 vaccine induced significant amounts of neutralizing antibodies, predominantly of the IgG2c isotype, and the virus was not detected in vaccine-challenged animals. Furthermore, monitoring blood glucose levels—and to a lesser extent, insulin antibodies—was found to be helpful in predicting vaccine responses. Taken together, our data suggest that the monovalent Mt10 vaccine has the potential to prevent infections caused by multiple CVB serotypes, as we have demonstrated in various pre-clinical models.

## 1. Introduction

The pathogenesis of type 1 diabetes (T1D) is fundamentally different from that of type 2 diabetes (T2D). While T2D in adults, is a metabolic disease resulting in insulin resistance, T1D generally occurring in adolescents, is an immune-mediated disease resulting in pancreatic β cell destruction, leading to insulin deficiency [1,2]. Although numerous therapeutic approaches have been tested experimentally [3,4], and several treatments are being used in the management of diabetic patients, no preventative strategies are currently available, partly due to the multifactorial nature of T1D [5].

T1D involves a strong genetic predisposition influenced mainly by human leukocyte antigen (HLA) class II genes, and to a lesser extent by HLA class I genes [6], but the concordance rate of developing T1D among identical twins and siblings is as low as ~30% and 6%, respectively [6,7,8]. This suggests that a combination of genetic and environmental factors such as infectious agents, drugs, dietary components, and gut microbiota may act in concert to trigger the disease in those affected [9,10,11,12]. Essentially, the prediabetic phase of T1D involves an autoimmune reaction causing the destruction of islet β cells accompanied by the appearance of antibodies to antigens such as insulin, glutamic acid decarboxylase, protein tyrosine phosphatase, Zinc Transporter 8 and cytoplasmic proteins in β cells [13]. Exposure to microbial infections during this disease-developing stage in genetically predisposed individuals is believed to trigger clinically noticeable disease resulting in the destruction of residual islet β cells as infections are established [5,14].

Of various microbial causes, a strong association exists between T1D and exposure to viruses, particularly enteroviruses such as Echoviruses, Coxsackievirus A and to a lesser extent, Rhinoviruses and Enterovirus 71 [15,16,17]. However, epidemiological, clinical and experimental data points to group B Coxsackieviruses (CVBs) as major triggers [6,18]. Six CVB serotypes, CVB1 to CVB6 that infect various organs such as the heart, pancreas, liver, central nervous, and gastrointestinal systems have been identified [19,20,21]. While all serotypes can cause pancreatitis, CVB3 is generally implicated in myocarditis [22,23,24], and CVB1 and CVB4 are known to be associated with insulitis, thus acting as cofactors for the development of T1D [25,26,27]. The reason for such differential disease phenotypes remains obscure since CVBs require Coxsackievirus-Adenovirus Receptor (CAR) to enter the target cells [28]. Importantly, endocrine cells of the pancreatic islets highly express CAR, providing an explanation for the tropism of CVBs [29,30]. Of note, identities between all six CVB serotypes are 76–80% and 86–91% at the nucleotide and amino acid levels, respectively. Thus, coordinated expression of various viral and host factors may determine damage to a specific tissue or cell type. It is also possible that the expression of specific isoforms of CAR may be necessary for different CVB serotypes to infect various cell types [31]. Nonetheless, the strong association between the occurrence of T1D and two CVB serotypes (CVB1 and CVB4) presents an opportunity to develop vaccines that may significantly impact the occurrence of T1D.

To that end, we made efforts to develop vaccines for CVBs and identified one live-attenuated CVB3 vaccine strain, designated mutant (Mt)10, which can prevent infections caused by homologous (CVB3) and heterologous (CVB4) strains [32,33]. As described elsewhere, the Mt10 virus vaccine prevented both myocarditis and pancreatitis by inducing neutralizing antibodies (nAbs) and antigen-specific T cell responses in challenge studies [32,33]. By extending these observations, we report here that the Mt10 vaccine virus could prevent CVB4-accelerated T1D in the non-obese diabetic (NOD) mice by inducing cross-reactive nAbs. As expected, however, the spontaneous progression of T1D in vaccinated mice was not altered.

## 2. Materials and Methods

### 2.1. Mice

Five-week-old female NOD mice (H-2^g7^) were procured from the Jackson Laboratory (Bar Harbor, ME, USA), maintained according to the institutional guidelines of the University of Nebraska-Lincoln, Lincoln, NE, and approved for animal studies by the university’s Institutional Animal Care and Use Committee. After one week of acclimatization, 6-week-old animals were used for experiments. Infection studies were performed according to biosafety level 2 guidelines, and euthanasia was performed using carbon dioxide as recommended by the Panel on Euthanasia of the American Veterinary Medical Association.

### 2.2. Virus Propagation and Titration

Mt10 vaccine virus, CVB1-conn5, CVB3 Nancy, and CVB4-E2 were propagated and titrated as described previously [32,33]. In brief, LLC-MK2 cells or Vero cells (ATCC, Manassas, VA, USA) were grown to ~90% confluency. LLC-MK2 cells were infected with CVB1-conn5, and Vero cells with CVB3 Nancy, CVB4-E2, and Mt10 vaccine virus. After cytopathic effects (CPEs) were confirmed, culture supernatants containing the virus were harvested, and viral stocks were stored in aliquots at −80 °C until further use. For viral titration, tissue culture infective dose (TCID)_50_ values were determined using the Spearman-Karber method [34].

### 2.3. Vaccination and Challenge Studies

For vaccine studies, the Mt10 virus stock was diluted in 1× phosphate-buffered saline (PBS) to contain 0.5 × 10^6^ virions/200 µL and administered intraperitoneally (IP) on days 0 and 14 (*n* = 15 mice). Control mice (saline recipients) received only 1× PBS (*n* = 15 mice). On day 21, vaccinated and control mice were divided into two groups each, and challenged with CVB4 (2000 TCID_50_/200 µL in 1× PBS) (*n* = 7 and *n* = 10 mice, respectively) or saline (*n* = 8 and *n* = 5 mice, respectively), IP. Groups of 3–5 mice were housed in filter-top cages assembled with closed-air circulation. Cages containing chow diet and waterers were changed every two days until termination of experiments. Animals had access to food and water *ad libitum* and were inspected twice daily; body weights were recorded every two days. An alternative food and fluid source, trans gel diet (ClearH_2_O, Portland, ME, USA), was placed on the cage floor as needed. Sera were collected on days 0, 14, and 21. At termination on day 56, sera, spleens, and pancreata were collected, for further analysis.

### 2.4. Blood Glucose and Diabetes Monitoring

Glucose levels were measured in blood collected from tail veins every week using a Contour Next ONE blood glucose meter (Ascensia Diabetes Care, Parsippany, NJ, USA). Mice with a blood glucose level of 300 mg/dL or above for two consecutive readings were deemed diabetic [35,36].

### 2.5. Histopathology

Harvested pancreata were fixed by immersion in 10% phosphate-buffered formalin. Tissues were sliced, and three representative 5 µm cross-sections were stained with hematoxylin and eosin (H. and E.). The sections were blinded to treatment and examined by a board-certified pathologist. The pancreatic lesions were evaluated for insulitis, peri-insulitis, peri-ductule inflammation, and fatty tissue replacement.

### 2.6. Virus Neutralization Assay

A virus neutralization test was performed using the sera obtained from different groups as described previously [32,33]. In brief, LLC-MK2 cells (for CVB1) or Vero cells (for CVB3 and CVB4) were plated at 0.25 × 10^6^ cells/mL in 96-well plates to obtain 90–100% confluency. Serum samples were heat-inactivated at 56 °C for 30 min, and two-fold serial dilutions were then made (1:40 to 1:40,960). Equal volumes of CVB1, CVB3, and CVB4 suspensions containing 100 TCID_50_/mL were incubated with serially diluted sera at 37 °C for 1 h in a humidified chamber with 5% CO_2_. After incubation, 100 µL of the mixture of each dilution was added in duplicates to plates containing monolayers of cells and incubated at 37 °C. After four days, plates were observed for CPE, and the highest serum dilution that showed protection from CPE was determined to be the neutralization titer, and the geometric mean titers (GMT) were calculated.

### 2.7. Determination of CVB-Reactive Antibodies

Serum samples from various groups (saline, Mt10 alone, CVB4 alone, Mt10 + CVB4) were collected on days 0, 14, 21 and 56, and analyzed for total immunoglobulins (Ig), IgM, IgG1, IgG2a, IgG2c, IgG3, IgA, and IgE as described previously [33]. Full-length CVB3 viral protein 1 (VP1) (GenScript, Piscataway, NJ, USA) and keyhole limpet hemocyanin (KLH) protein (Sigma-Aldrich, St. Louis, MO, USA) were procured commercially. Briefly, 96-well polystyrene microtiter plates were coated with or without CVB3 VP1 or an irrelevant control (KLH) (5 μg/mL) in 1× coating buffer and incubated at 4 °C overnight. The next day, plates were washed with a buffer (0.05% Tween^®^20 in 1× PBS) and blocked with an assay buffer (2% bovine serum albumin (BSA) and 5% normal goat serum in 1× PBS) for 1.5 h at room temperature (RT); serum samples prediluted in assay buffer (1:150) were then added to the plates in duplicates. After incubating at 37 °C for 1 h, plates were washed. For secondary antibodies, horseradish peroxidase (HRP)-labeled goat anti-mouse Ig, IgM, IgG1, IgG2a, IgG2c, IgG3, IgA, and IgE (Southern Biotech, Birmingham, AL, USA) were diluted in assay buffer (1:500) and plates were incubated at RT for 2 h, following which 100 µL of 1× tetramethylbenzidine solution (Rockland, Philadelphia, PA, USA) was added as substrate, and reactions were stopped using equal volumes of 1 M phosphoric acid. Plates were read at 450 nm using an automated enzyme-linked immunosorbent assay (ELISA) plate reader (BioTek Instruments, Winooski, VT, USA), and optical density (OD) values were measured.

### 2.8. Determination of Anti-Insulin Antibodies

Serum samples from various groups (saline, Mt10 alone, CVB4 alone, Mt10 + CVB4) collected on days 0, 21 and 56 were analyzed for anti-insulin antibodies as described previously [37]. High-binding, white, 96-well Immuno Plates (Thermo Fisher Scientific, Waltham, MA, USA) were coated with 100 µL of recombinant human insulin (Humulin R, Eli Lilly, Indianapolis, IN, USA) (10 µg/mL) in coating buffer, overnight. The plates were washed with a buffer (50 mM Tris in 0.2% Tween^®^20) and blocked for 2 h with an assay buffer (2% BSA in PBS) at RT. For competition with insulin, sera from different groups were diluted (1:10) with or without insulin (170 µg/mL) and incubated at RT. After 1 h, the serum-insulin mix was added to wells, and incubated for 2 h at RT. The plates were washed and allowed to dry. Next, a biotinylated anti-mouse IgG1 secondary antibody (clone RMG1-1, BioLegend, San Diego, CA, USA) was diluted in assay buffer (1:5000) and 100 mL was added to each well and incubated for 30 min at RT. After washing, the plates were incubated with europium-labeled streptavidin (DELFIA^®^ Eu-N1 Streptavidin, Perkin Elmer, Waltham, MA, USA) diluted in assay buffer (1:2000) for 15 min. The plates were washed a final time, and 150 µL of enhancement solution (DELFIA^®^, Perkin Elmer) was added to all wells and shaken for 10 min at 4 °C, in the dark. Finally, the plates were read using the Victor2 1420 Multilabel Counter (Perkin Elmer) at the rate of one well per second. For each sample, duplicates were averaged, and insulin competition wells (background) were subtracted from the non-competition wells (test) to obtain final readings.

### 2.9. Flow Cytometry

Splenocytes were harvested from groups of mice and transferred to a 96-well V-bottom plate. The following anti-mouse surface antibody markers (shown with clones, BioLegend) were used to stain the cells: CD19 (6D5, Alexa Fluor^®^ 488), B220 (RA3-6B2, Alexa Fluor^®^ 700), IgM (RMM-1, APC/Cyanine7), IgD (11-26c.2a, Pacific Blue™), CD95 (SA367H8, PE), GL7 (GL7, PE/Cy7) and CD4 (GK1.5, Brilliant Violet 421™). Cells were incubated in the dark on ice for 15 min and washed twice. For transcription factor staining, True-Nuclear™ Transcription Factor Staining Protocol was followed (BioLegend). Briefly, True-Nuclear™ 1× Fix Concentrate was added to the cells and incubated for 1 h in the dark at RT, after which cells were washed using True-Nuclear™ 1× Perm Buffer. This was followed by the addition of anti-mouse transcription factor-specific antibody, anti-Forkhead box P3 (FoxP3) (150D, Alexa Fluor^®^ 647), and incubation in the dark at RT for 30 min. Finally, cells were washed with perm buffer, and resuspended cells were acquired using the CytoFLEX LX flow cytometer (Beckman Coulter, Brea, CA, USA).

### 2.10. Cytokine Analysis

Serum samples obtained from groups of animals were evaluated for a panel of anti-viral cytokines and chemokines using the LEGENDplex™ Murine anti-viral kit (13-plex; BioLegend) and T helper (Th) cytokines using the LEGENDplex™ Murine Th cytokine panel kit (12-plex; BioLegend) [38,39]. The anti-viral panel included interferon (IFN)-γ, CXC chemokine ligand (CXCL)-1, tumor necrosis factor (TNF)-α, CC chemokine ligand (CCL)-2, interleukin (IL)-12p70, CCL-5, IL-1β, CXCL-10, granulocyte-macrophage colony-stimulating factor, IL-10, IFN-β, IFN-α, and IL-6, and the Th panel included IFN-γ, IL-5, TNF-α, IL-2, IL-6, IL-4, IL-10, IL-9, IL-17A, IL-17F, IL-22 and IL-13. Lyophilized cytokine standard mix provided in the kit was serially diluted in order to obtain standard curves. Capture beads/cytokine antibody conjugates were added to the diluted standards and test samples, followed by the addition of detection antibodies and streptavidin–phycoerythrin reagents. Samples were acquired by flow cytometry, and analyte concentrations were determined using the LEGENDplex™ data analysis software suite (BioLegend).

### 2.11. RNA Isolation and Real-Time Quantitative Polymerase Chain Reaction (RT-qPCR)

Pancreata were collected during termination and stored at −80 °C until RNA isolation. Approximately 30 mg of pancreatic tissue was homogenized with the FastPrep-96™ instrument as recommended (Lysing Matrix D 1.4 mm ceramic beads; MP Biomedicals, Irvine, CA, USA). Total RNA was isolated using the PureLink™ RNA Mini Kit (Invitrogen, Waltham, MA, USA), and samples were treated with deoxyribonuclease I and quantified using the NanoDrop™ ND-1000 spectrophotometer (Thermo Fisher Scientific). In a single-step reaction, RNA was reverse-transcribed, and PCR was performed using the PrimeTime™ One-Step RT-qPCR Master Mix (Integrated DNA Technologies, Coralville, IA, USA). Amplifications were performed using the CVB3 VP1 [target gene] (forward, 5′-TTGCATATGGCCCAGTGGAAG-3′; reverse, 5′-TGTGGATCCTTATTGCCTAGTAGTGGTAACTC-3′) and glyceraldehyde-3-phosphate dehydrogenase [GAPDH, housekeeping gene] (forward, 5′-CGGCAAATTCAACGGCACAGTCAA-3′; reverse, 5′- CTTTCCAGAGGGGCCATCCACAG-3′) TaqMan^®^ Gene Expression Assays (Applied Biosystems, Waltham, MA, USA) [40] and the CFX96 Touch Real-time PCR Detection System thermocycler (BioRad, Hercules, CA, USA). Expression of CVB3 VP1 was normalized to GAPDH using the 2^−(∆∆Ct)^ method as reported previously [33].

### 2.12. Statistical Analysis

Statistical analyses were performed using GraphPad Prism software v8.0 (GraphPad Software, Inc. La Jolla, CA, USA). Data sets pertaining to body weights, Igs, flow cytometry, RT-qPCR and cytokine bead array were analyzed using generalized least square model, unpaired Student’s t-test, Mann–Whitney U test and two-way ANOVA with Sidak’s post-test or Kruskal–Wallis test for pairwise comparisons between groups. Log-rank test with Bonferroni correction was used to analyze the statistical significance of the survival curves. Barnard’s exact test was used to examine the histological parameters.

## 3. Results and Discussion

We recently reported the creation of a novel live-attenuated CVB3 vaccine candidate, Mt10, that offers protection against both homologous (CVB3) and heterologous (CVB4) serotypes of CVB [32,33]. CVBs affect various organ systems [19,20,21], and although similar diseases are induced by multiple serotypes, their disease severities may vary in susceptible mouse strains such as A/J, BALB/c, or NOD mice [41,42,43]. For example, while CVB3 could induce severe myocarditis in A/J and BALB/c mice [44,45], pancreatitis could be induced to a comparable severity by CVB1, CVB3, and CVB4 [46,47,48]. Conversely, CVB4, despite infecting the heart tissue, has little myocarditis-inducing ability [32,48]. However, both CVB1, CVB4 and to a lesser extent CVB3 have been shown to trigger T1D in the NOD mouse model [41]. These variations appear to be due to the tissue tropism of different CVB serotypes [29,30]. For example, CVB4 infects pancreatic β cells, whereas the exocrine pancreatic acinar cells are infected by CVB3 [49]. Nonetheless, because of a high degree of similarity among all three serotypes (Table S1), there may be an induction of cross-reactive immune responses. Consistent with this prediction, our vaccine candidate, Mt10 vaccine virus bearing the CVB3 backbone, was found to induce robust cross-protective immune responses against CVB4, and to a lesser degree, CVB1 in A/J mice. However, the vaccine’s ability to influence T1D development in NOD mice was not investigated. Thus, we sought to test the hypothesis that the Mt10 vaccine could prevent accelerated T1D development triggered by diabetogenic CVB serotypes.

**Figure 1:**
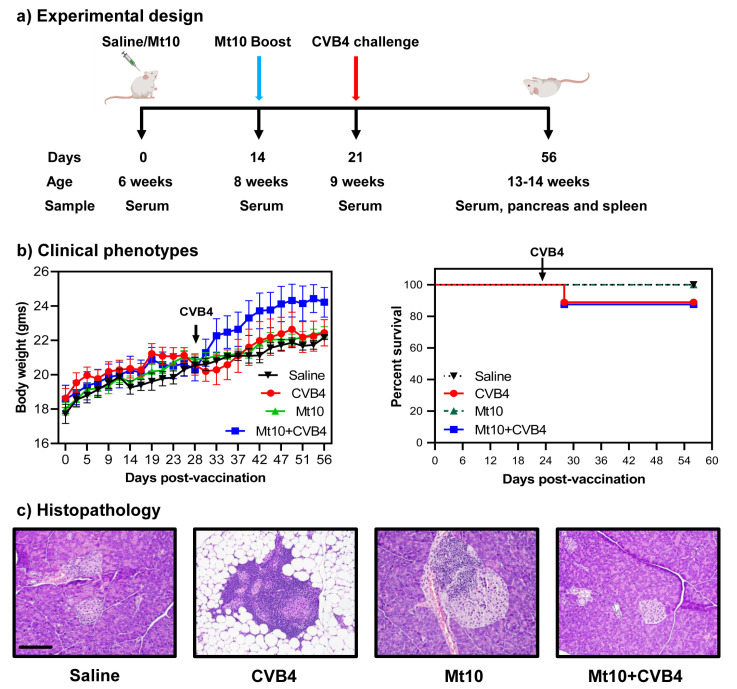
Mt10 vaccinated mice are protected from type 1 diabetes in challenge studies. (**a**) Experimental design. Groups of mice were given saline or were vaccinated with the Mt10 vaccine on days 0 and 14. After 7 days, CVB4 was administered to one group from each treatment. Experiments were terminated on day 56, and tissues were collected for histologic analysis. Serum was collected on days 0, 14, 21, and day 56 at termination. (**b**) Clinical phenotypes. Body weights (left panel) and survival rates (right panel) of different groups are shown. Generalized least square model was used to compare body weight changes in the saline, vaccine, and vaccine/challenged groups relative to the CVB4 group; log-rank test with Bonferroni correction was used to compare survival curves. (**c**) Histopathology. Pancreata preserved in 10% phosphate buffered formalin from the indicated groups were processed routinely and H. and E.-stained sections were evaluated for insulitis and other changes. Representative pancreatic sections are shown. Sections from the CVB4 group show destruction of islets of Langerhans and fatty tissue replacement whereas the samples from the saline and Mt10/CVB4-challenged groups shown are essentially normal. The Mt10 sample shows peri-insulitis and insulitis. Magnification, 20×; scale bar = 100 µm, applies to all panels. Data obtained from each group involve 5–10 mice.

To address our hypothesis, we chose CVB4 since the Mt10 vaccine virus could induce significant levels of cross-reactive nAbs to CVB4 in A/J mice [32]; and CVB4 was readily available for challenge studies. Of note, NOD mice begin to show inflammatory infiltrates with loss of pancreatic β cells around 8 weeks of age [50]. At about 12 to 15 weeks, these mice spontaneously develop insulitis and hyperglycemia [51,52], which can be aggravated to a greater severity upon exposure to CVB4 [27]. Furthermore, reports suggest that CVB infections can delay the diabetes process in young NOD mice, likely due to a non-specific viral effect on the murine immune system [53], whereas the same infections can accelerate the disease in older mice [41]. We used 6-week-old mice and immunized them with Mt10 twice, at 2-week intervals; 7 days later (equivalent to 9 weeks), animals were challenged with CVB4. At termination, animals had reached 13–14 weeks of age (Figure 1a). All animals gained weight over time, which is typical of healthy mice. Clinically, infected mice lost weight ~1-week post-infection and thereafter, but the weight loss was not substantial (Figure 1b, left panel). Likewise, none of the animals in any of the groups succumbed to disease. However, one mouse each in the CVB4 and Mt10 + CVB4 groups (~12%) died from virus infection (Figure 1b, right panel). Otherwise, animals in all groups appeared clinically normal during the 56-day observation period.

To further evaluate the disease-protective ability of the vaccine virus, we analyzed pancreata collected at termination for insulitis and other changes by H. and E. staining [33,54] (Figure 1c, Table 1). First, we noted mild insulitis in saline recipients (40%), which was an expected finding since NOD mice develop T1D spontaneously around 12–15 weeks of age [51,52]. Second, 89% of CVB4-infected mice had severe insulitis marked by the destruction of islets and the replacement by adipose tissue. Such an aggravation is consistent with previous reports that CVB4 infection could potentiate the progression of T1D [55]. Third, although insulitis was noted in 57% of vaccinated animals, vaccinated animals challenged with CVB4 had a significantly lower incidence of insulitis (13%) (Table 1). While a similar trend was noted with peri-ductule inflammation, the occurrence of peri-insulitis was not different across the groups, raising the question of whether pancreatic pathology could be correlated with the development of T1D and virus multiplication.

**Figure 2:**
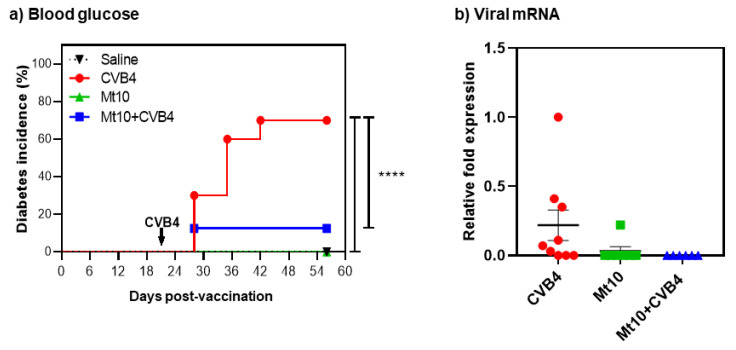
Mice vaccinated with Mt10 vaccine virus show enhanced protection against diabetes incidence. (**a**) Blood glucose. Blood glucose levels were monitored weekly, and diabetes incidence is shown. Mice with a blood glucose level of >300 mg/dL on two consecutive readings were deemed diabetic. Data sets obtained from each group involving *n* = 5–9 mice are shown. Log-rank test with Bonferroni correction was used to compare diabetes incidence. (**b**) Viral mRNA. Groups of mice were given saline or were vaccinated with Mt10 virus, and 7 days later were challenged with or without CVB4. Pancreata were collected 5 weeks post-challenge, total RNA was extracted, and viral RNA was estimated by RT-qPCR using VP1-specific nucleotide sequences. After normalizing the expression levels of viral RNA relative to GAPDH, 2^−(∆∆Ct)^ values were calculated. Mean ± SEM values representing 5–9 samples per group are shown. Mann–Whitney U test and one-way ANOVA with Tukey’s multiple comparison test was used to determine significance between groups. **** *p* ≤ 0.0001.

In this direction, we measured blood glucose levels once a week. We noted that none of the saline- or vaccine-recipients developed T1D (Figure 2a). In contrast, 70% of mice in the CVB4-alone group became diabetic, whereas only one mouse (12%) from Mt10 + CVB4 developed diabetes (Figure 2a). Additionally, we tested whether insulin antibodies followed a similar pattern. We chose to measure antibodies to insulin in our studies since they are commonly detected in T1D patients [56,57]. Nonetheless, standardizing insulin antibody assays in mice has been consistently debated [58,59,60,61], and this limitation was previously circumvented by developing a sensitive competitive europium insulin autoantibody assay [37]. Essentially, in this assay, because europium chelate has a longer fluorescence lifetime than traditional fluorophores, introducing competition for insulin greatly reduces the background signals that otherwise have been a technical hurdle with traditional insulin ELISAs [37]. The analysis revealed that only the CVB4-infected mice (at day 56) and 1 out of 5 vaccinated mice had elevated levels of insulin autoantibodies (Figure S1). In contrast, control mice and vaccinated mice challenged with CVB4 (at day 56) had relatively low levels of insulin antibodies, but no significant differences were noted between groups. Of note, it is common to evaluate at least two autoantibodies to determine the progression of T1D in clinical settings [7,62], which we have not done in our studies.

We next investigated if the vaccine-mediated protective effects are attributable to prevention of CVB4 infection by examining pancreata for viral RNA. The RT-qPCR analysis indicated that vaccinated mice as well as those vaccinated/challenged with CVB4 had negligible or no viral nucleic acids in their pancreata (Figure 2b). A relatively increased expression of viral RNA was detected in mice infected with CVB4 (Figure 2b), but the differences were not significant. This data suggest that virus persistence may not be necessary to accelerate the disease process and that by preventing infection, the vaccine virus might have suppressed the development of T1D in challenged animals.

**Figure 3:**
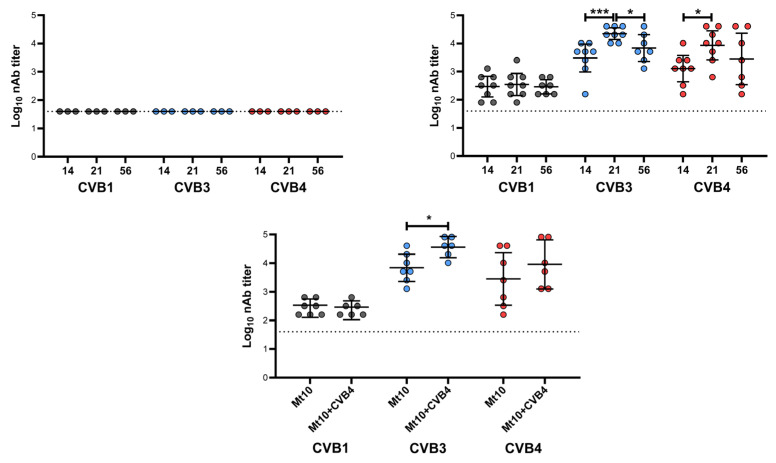
Mt10 vaccine induces nAbs against CVB4. Sera were collected from recipients of saline (**top left**), Mt10, (**top right**) and Mt10 + CVB4 (**bottom**) on days 14, 21, and 56. Two-fold serial dilutions from 1:40 up to 1:40,960 were made, and samples were incubated with CVB1, CVB3, or CVB4. The mixtures were later transferred to plates containing monolayers of LLC-MK2 cells for CVB1 or Vero cells for CVB3 and CVB4 and incubated for 4 days to calculate the percentage neutralization based on CPE. The top left panel indicates the nAb titers determined against CVB1, CVB3, and CVB4 in the saline group that served as a baseline. The top right panel indicates the nAbs determined in the Mt10 vaccine group against CVB1, CVB3, and CVB4 at three different time points, and the profiles were compared. The bottom panel indicates the nAb titers determined in the Mt10 vaccinated/CVB4 challenged group and the profiles were compared with those of Mt10 vaccine group at termination (day 56). Data from *n* = 3–8 samples are shown. Two-way ANOVA with Sidak’s post-test was used to compare significance of nAb titers. * *p* ≤ 0.05, *** *p* ≤ 0.001.

To mechanistically evaluate whether vaccine-mediated T1D protection might be due to induction of nAbs, we performed a live virus neutralization assay on sera collected before and after vaccination (at days 14, 21 and 56; Figure 3). Expectedly, none of the animals in the control group had any detectable nAbs for any of the CVB serotypes tested (Figure 3, top left panel). Similar analysis revealed that mice vaccinated with Mt10 had nAbs against CVB1, CVB3, and CBV4 in the decreasing order of CVB3/CVB4, and CVB1 (Figure 3, top right panel). Of note, nAb levels for CVB1 were similar across all time points in vaccinated mice with GMTs ranging from 289 to 343, whereas nAb levels for CVB3 reached a GMT of 3044 post-prime, and peaked on day 21 (post-boost) with a GMT of 22,334, and reached closer to the pre-boost steady levels (GMT 6891) by day 56 (Figure 3, top right panel). Consistent with our previous observations [32], the high nAb titers against CVB4 associated with Mt10 vaccine peaked at the post-boost time point (day 21) with a GMT of 8611 and were stably maintained thereafter (GMT 2827) (Figure 3, top right panel). We next compared the nAb titers between vaccinated and vaccinated/CVB4-challenged groups at day 56, leading us to note that the titers of nAbs for CVB1 remained similar in both groups with a GMT of 290 for vaccinated and GMT of 253 in CVB4-challenged group (Figure 3, bottom panel). Further analysis revealed a significant increase in nAbs for CVB3 (GMT 36,491) in the CVB4-challenged group (Figure 3, bottom panel), indicating that CVB4 could increase the cross-reactive vaccine response to CVB3. Although such a trend existed for CVB4 (GMT 9123) in the challenged group, the differences in nAb titers were not significant between vaccinated and challenged groups (Figure 3, bottom panel). Yet, it was critical to determine the nature of antibody isotypes produced in each group.

**Figure 4:**
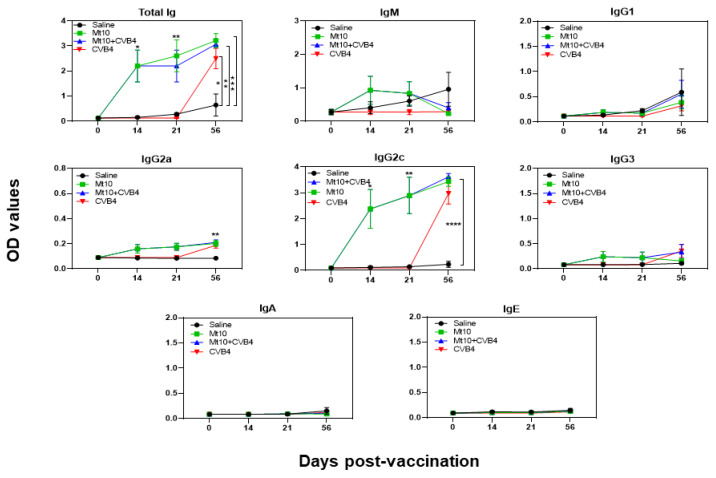
Antibody responses induced by Mt10 vaccine predominantly included the IgG2c subclass. Sera collected from the indicated groups at various time points were diluted (1:150) and added in duplicates to high-binding plates previously coated with CVB3-VP1 or KLH (control). After adding HRP-conjugated goat anti-mouse total Ig, IgM, IgG1, IgG2a, IgG2c, IgG3, IgA, and IgE as detection antibodies, reactions were stopped. Plates were read at 450 nm to obtain the OD values. Mean ± SEM values obtained from *n* = 5–8 samples are shown. Two-way ANOVA and Mann–Whitney U test were used to determine significance between groups. * *p* ≤ 0.05, ** *p* ≤ 0.01, *** *p* ≤ 0.001, and **** *p* ≤ 0.0001.

We evaluated the antigen specificity of antibodies generated against CVB4 by using the VP1 of CVB3 and KLH as positive and control antigens, respectively, as described previously [32,33], since a high degree of similarity (~90%) exists between VP1s of CVB3 and CVB4. We made two major observations using the sera collected on days 0, 14, 21, and 56 (Figure 4). (i) Total Ig levels were significantly elevated in both the Mt10 (*p* ≤ 0.001) and Mt10 + CVB4 groups (*p* ≤ 0.01) as compared to the saline group (Figure 4). The Ig levels persisted all through the 56-day experimental period. Likewise, animals in the CVB4-alone group revealed significantly elevated levels of Ig (*p* ≤ 0.05). These data suggest that the NOD mice could respond to multiple CVB serotypes and generate immune responses. (ii) Patterns similar to those described above were noted for IgG2c and, to a lesser extent, IgG2a (Figure 4). Otherwise, no alterations were noted in other isotypes (IgM, IgG1, IgG3, IgA, and IgE). Thus, we concluded that vaccine-induced protection might have occurred through the generation of predominantly IgG2c nAbs cross-reacting with CVB4. This protective ability of the vaccine may involve the mediation of virus-specific T cell responses since T cell cytokines are critical for isotype switching. For example, IFN-γ promotes IgG2c [63,64]. In that direction, we tested for various anti-viral (Figure S2) and T helper subsets (Figure S3) cytokines/chemokines in the serum samples collected on days 0 and 56. However, none of the cytokines tested showed any significant differences between groups. It is possible that cytokine analysis in the blood compartment at the late time points is unlikely to yield informative data. We drew similar conclusions by evaluating various B cell subsets in the splenocytes, such as B cells (CD19^+^B220^+^), class-switched B cells (IgM^−^IgD^−^), and germinal center cells (CD95^+^CL7^+^) (Figure S4), including the possibility that Mt10 vaccine could induce the regulatory T (Treg) cells (CD4^+^FoxP3^+^) because CVBs have been shown to induce Treg cells [65,66,67]. The analysis revealed no major differences between groups, except that the Ig class-switched cells tended to occur more in the CVB4-alone group, followed by the vaccine and vaccine/CVB4-challenged groups. Likewise, an increasing trend was noted in the vaccine group for the frequencies of Treg cells when compared to the saline group [*p* = 0.06] (Figure S4). Taken together, our data suggest that cross-reactive nAbs may have led to the prevention of CVB4 infection, thus contributing to vaccine-induced protection against CVB4-accelerated T1D. This notion is supported by the finding that the CVB4-alone group had exacerbated T1D (Figure 2).

Given the importance of T1D, the use of vaccines to prevent the disease has been discussed in the literature [68,69,70,71], but questions may arise as to which viruses can be targeted and how, what the target population for the vaccine is if vaccines become available, and what the impact might be. There are no easy answers to these questions, primarily because T1D has been associated with a variety of viruses [18,72]. Based on serological evidence, however, the major candidates appear to be CVBs at a global level [73,74]. Furthermore, although it is hard to prove the cause-and-effect relationship in humans, CVBs could cause diabetes in monkeys [75], and studies from the pre-clinical NOD model suggest that CVBs could trigger T1D in genetically susceptible individuals [52]. In support of this proposition, enteroviruses and their signatures (antibodies or nucleic acids) have been detected in T1D patients, including genetically at-risk infants [76,77,78]. Thus, a suggestion has been made that vaccines may prevent the initiation of infections when given to genetically susceptible individuals at an early age prior to the start of the β cell damaging process, which would limit T1D development in vaccine recipients [79,80]. However, accumulated literature suggests that T1D can be seen in patients without previous T1D history, implying that vaccinating broader populations may be appropriate [81,82]. Furthermore, infection of β cells with viruses can occur at any age, and T2D patients could have T1D as enteroviruses have been detected in T2D patients at a higher rate than in unaffected controls [15,83,84]. It has also been proposed that vaccine viruses may induce disease-protective Treg cells, as shown in NOD mice exposed to CVB infections [65,66]. Therefore, the use of anti-viral vaccines has been estimated to prevent at least 50% of T1D cases [18], but questions arise as to their desired characteristics.

It is not practical to generate serotype-specific vaccines to prevent T1D since multiple CVB serotypes can trigger the disease [6,18]. For having successfully developed effective vaccines against polioviruses [15,85] and enterovirus 71 [70,86], generation of similar vaccines for T1D in relation to CVBs is possible. In that direction, formalin-inactivated non-adjuvanted monovalent CVB1 vaccine was shown to prevent CVB1 infection and also CVB1-induced T1D development in NOD mice and the transgenic mice expressing suppressor of cytokine signalling-1 on the NOD background [87]. Likewise, formalin-inactivated whole CVB4-E2 was shown to delay the onset of T1D in NOD mice, but autoantibodies to several beta-cell antigens were elevated potentially resulting from cross-reactivity [88]. Recently, a hexavalent inactivated vaccine involving all six serotypes of CVB has been tested in mice and nonhuman primates [89]. Although this vaccine was effective in preventing CVB infections by producing nAbs, antigen specificity and characterization of antibody isotypes were not investigated. Our vaccine is a live attenuated virus able to induce both antibody and T cell responses. While we have demonstrated the former, we could not analyze antigen-specific T cell responses in NOD mice due to a lack of relevant tools. However, the possibility remains that virus-reactive T cells can be generated in vaccine recipients because antibody production—as expected in NOD mice—was almost entirely skewed toward the IgG2c response that requires IFN-γ from T cells [63,64]. Conversely, CVBs are RNA viruses and therefore prone to mutations [90,91], so it is possible that the live vaccine viruses may revert to virulence. To overcome this possibility, we are generating virus-like particles (VLPs) from Mt10 with the expectation that their structural proteins could induce protective responses similar to live Mt10 virus. In support of this notion, the VLP-based vaccine for CVB3 was shown to induce high nAbs in mice, but its ability to prevent T1D development was not investigated [92].

In summary, we have demonstrated that the monovalent Mt10-CVB3 vaccine virus could protect against CVB4-accelerated T1D in NOD mice. Since multiple CVB serotypes could induce similar organ-specific diseases, as is the case with CVB1 and CVB4 in the development of T1D [25,26,27], the Mt10 vaccine has the potential to produce cross-reactive nAbs for other serotypes. We had previously demonstrated that the Mt10 vaccine could prevent myocarditis induced by CVB3, as well as pancreatitis induced by both CVB3 and CVB4 in A/J mice [32,33]. Thus, we expect that the Mt10 vaccine could be used to prevent infections caused by multiple CVB serotypes that induce different organ-specific diseases. While such investigational studies in the mouse models provide a proof of concept, for a greater validation, vaccine efficacies are to be tested in the non-human primate models of T1D [93,94].

## Figures and Tables

**Table 1:** Histological evaluation of pancreata in saline, CVB4, Mt10-infected, and Mt10-infected/challenged mice.

Parameters	Saline	CVB4	Mt10	Mt10 + CVB4
Insulitis	2/5 (40.0)	8/9 (88.9)	4/7 (57.1)	1/8 (12.5) *
Peri-insulitis	3/5 (60.0)	7/9 (77.8)	6/7 (85.7)	7/8 (87.5)
Peri-ductule inflammation	5/5 (100.0)	7/9 (77.8)	4/7 (57.1)	2/8 (25.0)
Destruction/Fatty replacement	0 (0.0) *	8/9 (88.9)	2/7 (28.5)	1/8 (12.5) *

* Represents *p* < 0.05 by Fischer’s exact test (two-sided) comparing CVB4 group with other groups.

## Data Availability

Not applicable.

## Supplementary Materials

The following supporting information can be downloaded at: https://www.mdpi.com/article/10.3390/vaccines11010076/s1, Table S1: Comparison of sequences between different CVB serotypes; Figure S1: Serum from Mt10 vaccinated mice, challenged with CVB4 did not reveal insulin autoantibodies; Figure S2: Analysis of anti-viral cytokines and chemokines in sera from animals vaccinated with Mt10 vaccine virus and challenged with CVB4; Figure S3: Analysis of T helper cytokines in sera from animals vaccinated with Mt10 vaccine virus and challenged with CVB4; Figure S4: Immunophenotyping of splenocytes obtained from animals vaccinated with Mt10 vaccine virus and challenged with CVB4.
