# Supplementary material for: A Monovalent Mt10-CVB3 Vaccine Prevents CVB4-Accelerated Type 1 Diabetes in NOD Mice
Source: Vaccines (Basel). 2022 Dec 29;11(1):76. doi: 10.3390/vaccines11010076 (PMC9864234; doi:10.3390/vaccines11010076)
Supplement: Supplementary file 1 [file vaccines-11-00076-s001.zip › Table S1.pdf]

Table S1: Comparison of sequences between different CVB serotypes.

Sequences	GenBank accession numbers	Percentage similarity matrices*					
		CVB1	CVB2	CVB3	CVB4	CVB5	CVB6
Nucleotide	Coxsackievirus B1| gb|M16560.1|CXA1G:1-7389		78.34	80.46	77.06	79.19	78.30
	Coxsackievirus B2 strain Ohio-1|gb|AF085363.1|:1-7411	78.34		79.37	77.34	78.90	78.02
	Coxsackievirus B3 strain Nancy |gb|JX312064.1|:1-7399	80.46	79.37		76.74	79.54	77.68
	Coxsackievirus B4 strain E2 |gb|AF311939.1|:1-7397	77.06	77.34	76.74		76.27	77.05
	Coxsackievirus B5 strain Faulkner|gb|AF114383.1|:1-7400	79.19	78.90	79.54	76.27		77.53
	Coxsackievirus B6 strain Schmitt|gb|AF105342.1|:1-7398	78.30	78.02	77.68	77.05	77.53	
Amino acid	Coxsackievirus B1|gb|AAC00531.1|:1-2182		89.27	89.53	88.47	91.23	90.69
	Coxsackievirus B2 strain Ohio-1|gb|AAD46138.1|AF081485_1:1-2187	89.27		87.23	88.63	88.69	88.96
	Coxsackievirus B3 Nancy|gb|AAA74400.1|:1-2185	89.53	87.23		86.52	89.50	88.59
	Coxsackievirus B4 strain E2 |gb|Q86887.3|POLG_CXB4E_1:1-2183	88.47	88.63	86.52		88.48	88.90
	Coxsackievirus B5 strain Faulkner|gb|AAF21971.1|AF114383_1:1-2185	91.23	88.69	89.50	88.48		91.31
	Coxsackievirus B6 strain Schmitt|gb|AAF12719.1|AF105342_1:1-2184	90.69	88.96	88.59	88.90	91.31	

* Percentage sequence similarity between serotypes was calculated using the MUSCLE alignment algorithm
