# Supplementary figures and images for: A Monovalent Mt10-CVB3 Vaccine Prevents CVB4-Accelerated Type 1 Diabetes in NOD Mice
Source: Vaccines (Basel). 2022 Dec 29;11(1):76. doi: 10.3390/vaccines11010076 (PMC9864234; doi:10.3390/vaccines11010076)

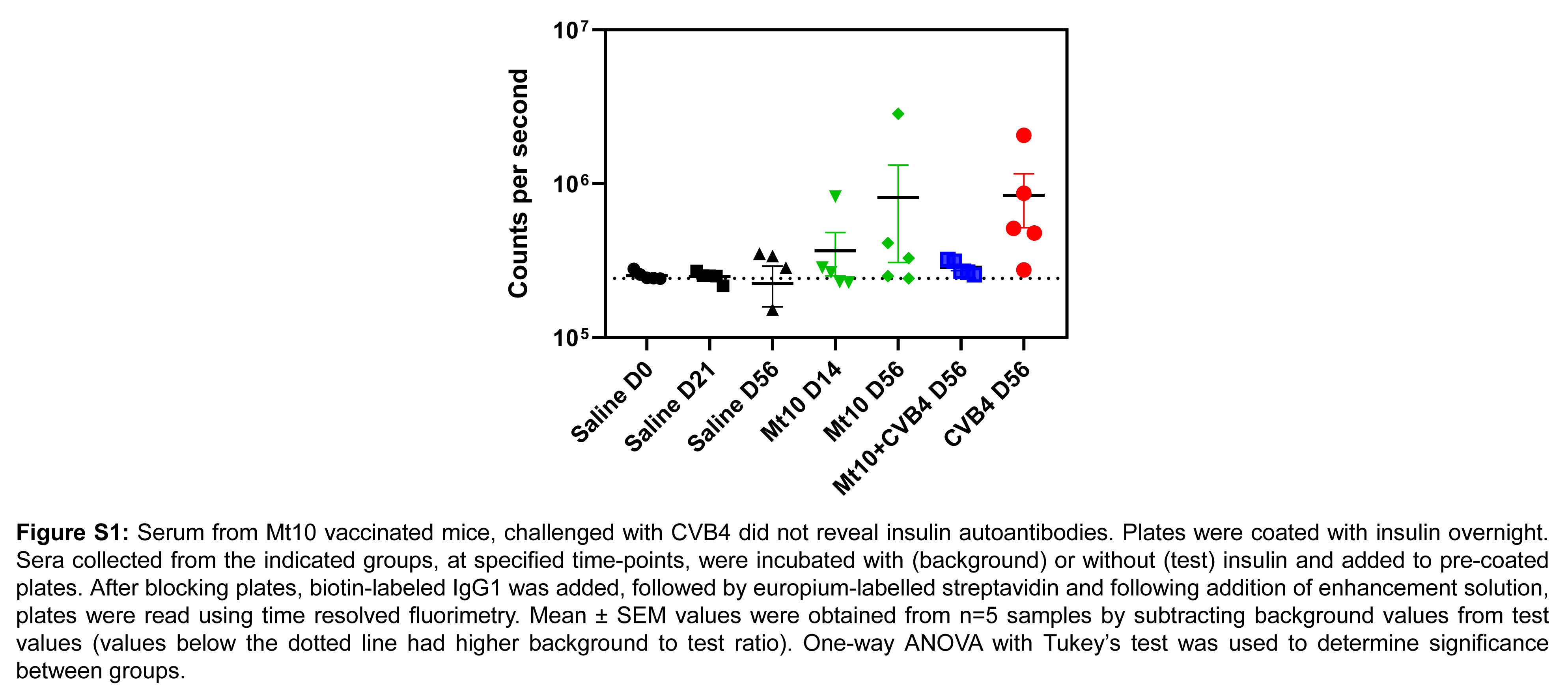

Supplement: Supplementary file 1 [file vaccines-11-00076-s001.zip › Figure S1.tif]

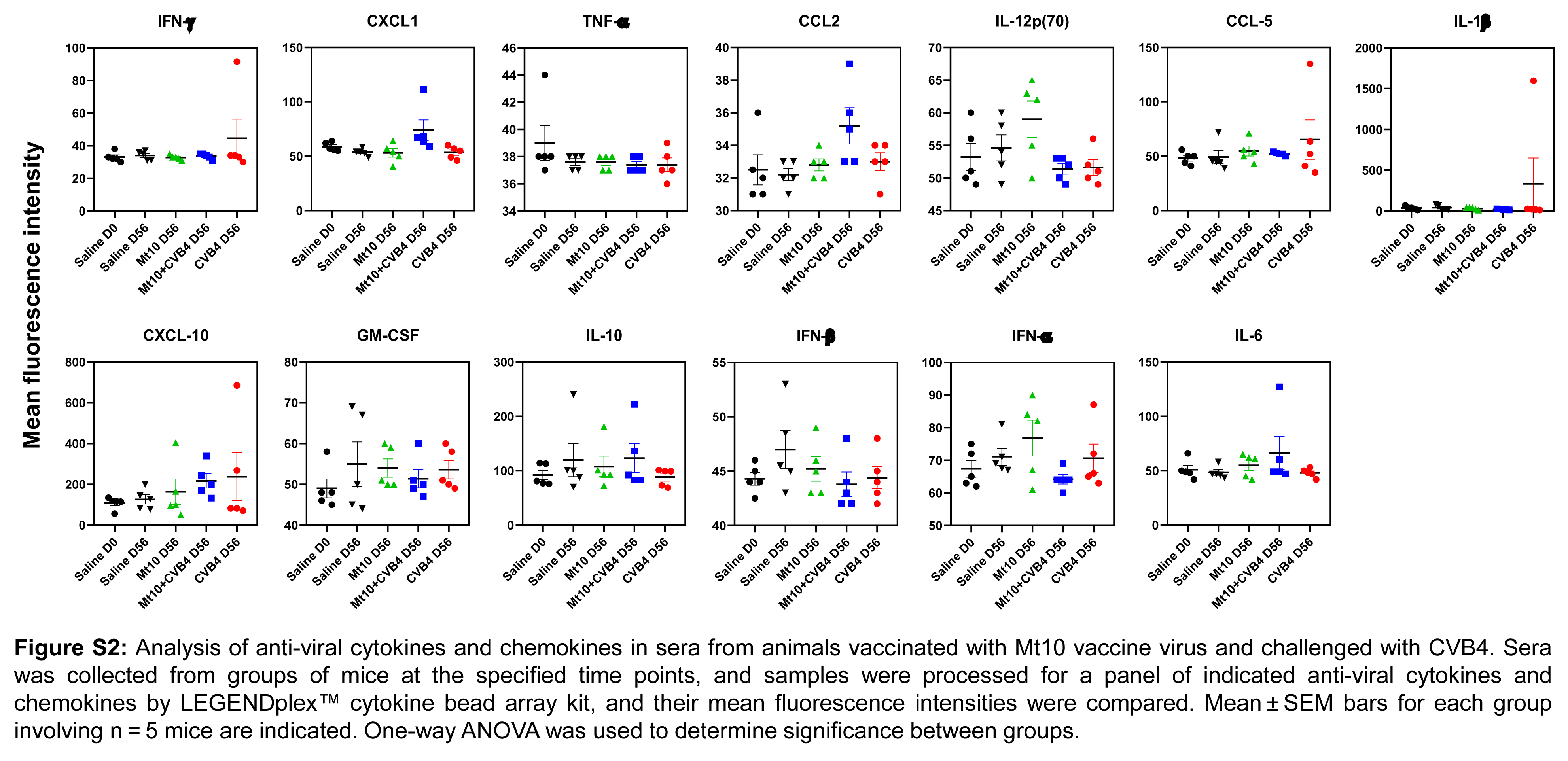

Supplement: Supplementary file 1 [file vaccines-11-00076-s001.zip › Figure S2.tif]

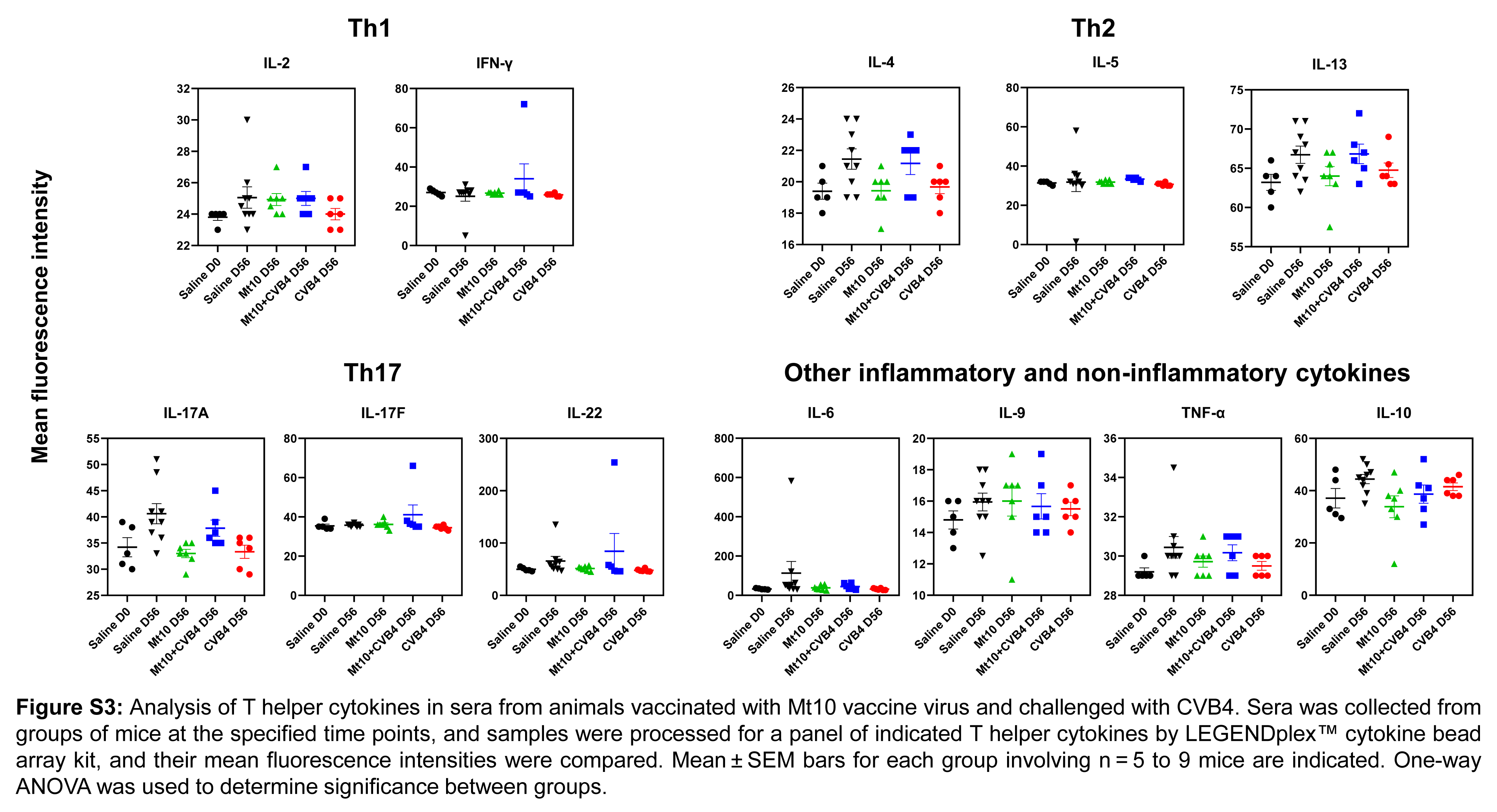

Supplement: Supplementary file 1 [file vaccines-11-00076-s001.zip › Figure S3.tif]

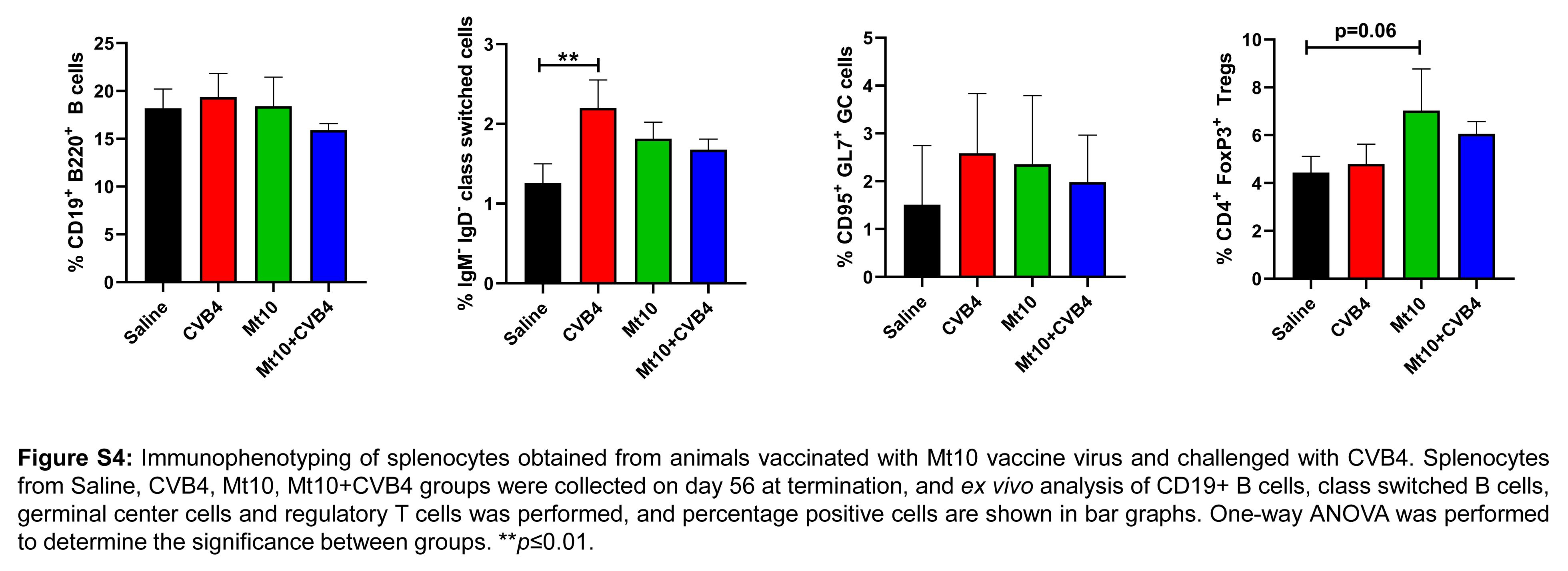

Supplement: Supplementary file 1 [file vaccines-11-00076-s001.zip › Figure S4.tif]
